# Interpersonal Associations Between Affect and Depressive Symptoms in Persons With Early Dementia and Their Spouses

**DOI:** 10.1093/geroni/igab046.1152

**Published:** 2021-12-17

**Authors:** Joan Monin, Amanda Piechota, Sumaiyah Syed

**Affiliations:** 1 Yale School of Public Health, New Haven, Connecticut, United States; 2 Yale School of Public health, New Haven, Connecticut, United States

## Abstract

Positive and negative affect have independent effects on health and occur frequently in close relationships. No research to our knowledge has examined self-reported affective experiences of persons with dementia (PWD) and their spouses and interpersonal associations with their psychological health. Secondary analysis of baseline interviews from a randomized clinical trial testing a stress reduction intervention in 45 couples (n=90) examined whether individuals’ positive and negative affect were associated with their own depressive symptoms (actor effects) as well as their partner’s depressive symptoms (partner effects) and whether these associations differed for PWD and spouses. Actor partner interdependence model results showed that for PWDs and spouses, one’s own positive affect was related to one’s own lower depressive symptoms (B=-3.10, SE=.59, df=58.70, p<.001), and one’s own negative affect was associated with one’s own greater depressive symptoms (B=6.62, SE=.60, df=65.67, p<.001). These effects were independent from each other. Partner effects were not significant.

